# The Influence of Family Intervention on the Treatment of Adolescent Patients With Borderline Personality Disorder: A Literature Review

**DOI:** 10.7759/cureus.40758

**Published:** 2023-06-21

**Authors:** Jingxiong Pu, Maheen F Zaidi, Maithily Patel, Lakshmi Malvika Atluri, Natalie A Gonzalez, Navya Sakhamuri, Sreekartthik Athiyaman, Bhawna Randhi, Sai Dheeraj Gutlapalli, Lubna Mohammed

**Affiliations:** 1 Psychiatry and Behavioral Sciences, California Institute of Behavioral Neurosciences & Psychology, Fairfield, USA; 2 Medical College, Aga Khan University Hospital, Karachi, PAK; 3 Research, California Institute of Behavioral Neurosciences & Psychology, Fairfield, USA; 4 Family Medicine, California Institute of Behavioral Neurosciences & Psychology, Fairfield, USA; 5 Surgery, Dr. Pinnamaneni Siddhartha Institute of Medical Sciences and Research Foundation, Vijayawada, IND; 6 General Surgery, California Institute of Behavioral Neurosciences & Psychology, Fairfield, USA; 7 Pediatrics, Medical University of Graz, Graz, AUT; 8 Pediatrics, California Institute of Behavioral Neurosciences & Psychology, Fairfield, USA; 9 Internal Medicine, California Institute of Behavioral Neurosciences & Psychology, Fairfield, USA; 10 Medicine, NRI Medical College, Chinakakani, IND; 11 Medicine, California Institute of Behavioral Neurosciences & Psychology, Fairfield, USA

**Keywords:** psychotherapy, family intervention, family therapy, family, borderline personality disorder

## Abstract

Borderline personality disorder (BPD) is a widespread mental disorder linked to functional impairment and a high suicide rate. Adolescent BPD is now recognized as a reliable and valid diagnosis in psychiatric classification systems and national treatment guidelines. Family issues, such as parental underinvolvement or neglect, may affect the mentalization process and attachment styles. Thus, the family is crucial to understanding the etiology of BPD in adolescents. Family intervention was primarily used as a component of the psychotherapy strategy in the current treatment of BPD, including pharmacological and psychotherapy measures. The primary objective of this study is to review previous research on the effectiveness of family intervention in treating adolescents with BPD. Although there is currently little data, studies in this paper show that family intervention is a realistic treatment option for adolescents with BPD.

## Introduction and background

Borderline personality disorder (BPD) is a mental disorder marked by unstable interpersonal relationships, self-image, affect, and impulsivity that begins in early adulthood [1]. Studies indicate that BPD can be reliably diagnosed in adolescents [2], and the Diagnostic and Statistical Manual of Mental Disorders (DSM-5) concurs [1]. The distribution of knowledge about BPD in youth (adolescents and emerging adults) during the previous two decades has provided a strong foundation for early diagnosis and treatment ("early intervention") for BPD and subthreshold borderline personality illness [3]. BPD in adolescents displays the same or even higher prevalence rates as that of the adult population [4]. It is a leading cause of disability-adjusted life years in young people and a significant financial burden for their families [3]. 

Adolescence itself is marked by "BPD features" of high affect instability, increased aggression, impulsivity, and identity issues, as well as a normal rise in maladaptive personality traits, self-harm, and other behaviors that correlate with the fundamental criteria of BPD [4,5]. 

Several etiological theories suggest that BPD develops as a result of interactions between a child's preexisting emotional vulnerability and adverse family circumstances, particularly in the context of negative interpersonal interactions between the child and caregivers [6,7]. Adverse family backgrounds and suboptimal parenting could predict early BPD symptoms at the age of 11 [8,9]. Family therapy concentrates on the systemic background of problems, with therapists attempting to comprehend family structures and the often-invisible but influential dynamics entrenched within the family system [10]. Family structure impacts the regulation of interpersonal boundaries and interactions between family members. People with BPD frequently experienced traumatic neglect or grew up in a chaotic/dysfunctional family environment during their formative years [11] with limited opportunity to establish healthy boundaries and relationships. Family intervention can strengthen positive alliances between the medical team and family members of patients with BPD [8]. Here, the use of psychotherapy with family therapy is encouraging, particularly for adolescents with BPD [10]. 

The purpose of this study was to examine the use of family therapy and family intervention on adolescent BPD patients. This study is timely because the value of working with the family in the treatment needs to be emphasized, and evidence for the additional effects of family intervention is needed [12]. 

Search strategy

Detailed research was conducted using the keywords and Medical Subject Headings (MeSH) terms in Table 1 to include the studies that analyzed and assessed the effect of family intervention on adolescent patients. As family intervention is emphasized in various psychotherapies such as dialectical behavioral therapy (DBT) and mentalization-based therapy (MBT) [13], articles regarding psychotherapy treatment of adolescent BPD patients were also included. All the articles considered were chosen without the restriction of gender, ethnicity, demographical limitations, and publication type. We included studies published between 2012 and 2022. All the articles chosen were in English. This article did not follow the Preferred Reporting Items for Systematic Reviews and Meta-Analyses (PRISMA) guidelines as it is a traditional review article. The last date of our search was June 16, 2022. 

**Table 1:** Search Strategies and the Number of Results Identified From the Search MeSH = Medical Subject Headings

Databases	Keywords	Search Strategy	Filters	Results
PubMed	Borderline personality disorder; family intervention; family therapy; adolescent;	#1 (Borderline personality disorder [Title/Abstract]) OR (Borderline personality disorder [MeSH Major Topic]) #2 ((((Family therapy [MeSH Major Topic]) OR (family therapy [Title/Abstract])) OR (family involvement [Title/Abstract])) OR (family[MeSH Major Topic])) OR (family [Title/Abstract]) #3 (((Adolescent* [MeSH Major Topic]) OR (adolescent* [Title/Abstract])) OR (Teenager* [Title/Abstract])) OR (Youth [Title/Abstract]) #4 #1 AND #2 AND #3	Full text, in the last 10 years, English, Adolescent: 13-18 years	56
PsycInfo	Borderline personality disorder; family therapy; adolescents	DE “Borderline Personality Disorder” OR “borderline personality” AND DE “family therapy or family counseling or family intervention or family systems therapy”	Year：2012-2022, Language: English, Age: Adolescent (13-17 years）	531

## Review

Discussion

This section discusses the etiology, diagnosis, extant treatment approaches, and the present literature on the usage of family intervention of BPD in adolescent patients. Various review articles, quantitative and qualitative studies, and trials will be summarized. 

Etiology

Studies suggest that BPD results from the interaction between constitutional vulnerabilities and parental underinvolvement or neglect [14]. It results in deficiencies in the child's mentalization-based ability to regulate emotions. The invalidating atmosphere may also impede attachment styles and the acquisition of emotional regulation strategies [2]. Even more so for parents with these genetic predispositions, temperamental factors, such as emotional reactivity or difficulty being comforted, can be challenging.

There is a strong association between BPD and insecure (mostly preoccupied) attachment, according to the majority of studies examining the attachment style of BPD patients [15,16]. Affective instability and unstable representations of attachment figures characterize preoccupation. As a result, patients anticipate that they cannot rely on others for assistance. Adolescent BPD risk factors include a history of disrupted attachment, maternal neglect, maternal rejection, grossly inappropriate parental behavior, number of mother and father surrogates, physical and sexual abuse, parental loss [17], perceived caregiver criticism [18], and low socioeconomic status [19]. These elements lend support to an insecure attachment etiological model. 

An expert research review [12] points to the fact that inherited and environmental risk factors contribute independently and interactively to the etiology of BPD, which suggests an interaction between genetic risk factors and abuse in the family. When any of these are present, they all increase the risk, as does a family history of mental illness. 

Diagnosis 

Diagnosing BPD in adolescents is justifiable and feasible [8] and is supported by DSM-5 [1]. To date, the major diagnostic classification systems have not adopted BPD criteria with a developmental focus. Therefore, adult BPD criteria are applied to adolescents [1]. BPD can present with a myriad of symptoms, including identity disturbance, self-injurious behavior, impulsivity, fear of abandonment, marked reactivity of mood, and chronic feelings of emptiness [1].

Early detection and treatment are expected to reduce chronicity and adverse health outcomes. Increasing knowledge and decreasing stigma among professionals will likely promote early detection and lead to more timely and targeted interventions to reduce impairment and improve the prognosis for adolescents with BPD [8]. When diagnosing adolescents, differences between adults and adolescents must be considered. Due to the overrepresentation of acute symptoms in adolescent BPD [8], acute mental crises that may occur during a mental state disorder or a developmental crisis must be distinguished from characteristics that indicate more general maladaptive and dysfunctional behaviors.

Current Management Approaches 

Patients with BPD are typically treated with medications to manage their mood, anxiety, agitation, self-harm, and emotional dysregulation, despite inadequate proof of efficacy and potential adverse effects associated with the medication prescribed [10]. Medication might bring temporary control, safety, and relief, but evidence of effectiveness is limited and may bring unwanted side effects. Additionally, medication does not address the etiological factors that may contribute to poor outcomes, further disempowerment, regression, or damage. Models of effective psychotherapy include DBT, MBT, cognitive analytic therapy, transference-focused psychotherapy, and emotion regulation therapy. They may improve the psychological health, behavior, and resiliency of individuals [10].

Importance and Role of Family Intervention

**Figure 1:**
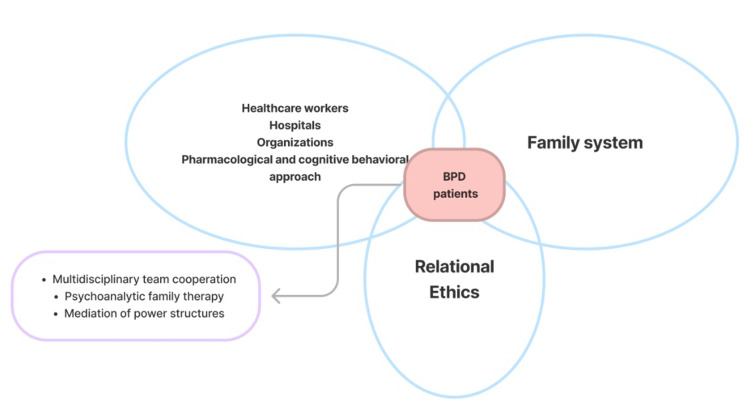
Key Constructs of the Dynamic Interactions Between the Patient, Family, Healthcare System, Methods of Treatment, and Ethical Decision-Making Adapted from: Choi, 2018 [10], with permission from the publisher. BPD = borderline personality disorder

The care of BPD patients stands at the intersection of medical care, family system, and relational ethics, as illustrated in Figure 1. 

In family therapy, the systemic backdrop of issues is the main focus, and therapists work to comprehend family dynamics and the sometimes invisible but often important family structures. Family is a valuable ally as an information source, the primary support for the adolescent, and a crucial component in managing adolescent BPD. Additionally, if the family environment interacts with the genetic vulnerability of this disorder, a family-level intervention may be protective. BPD symptoms in adolescents have tremendous effects on their families, especially their mental health, which must be validated [20]. Studies have shown that the severity of BPD symptoms correlates with the intensity of parental burden [21,22]. Family treatment is encouraged in BPD treatments [22], as demonstrated in Good Psychiatric Management for BPD [23], where family involvement such as a family psychoeducation group, “Family Connections” multifamily group, or conjoint sessions with family is emphasized [23]. Similar to this, parenting techniques and family psychoeducation training are suggested as potential early therapies for those with subthreshold characteristics of BPD or presenting the first episode of threshold BPD [24]. 

Many studies have included family intervention in treating BPD in adolescents. Family intervention is often included as a part of psychotherapy programs, especially DBT programs. It is crucial to actively involve families in DBT treatment. Their participation in the multi-skills training group may enable them to acquire the skills necessary to comprehend their own and their children's actions while serving as role models and coaches for them, which may contribute to the generalization and maintenance of such skills [2]. Parents of teenagers with BPD symptoms or a diagnosis may benefit from dialectical behavior treatment for adolescent therapy family skills training; it provides them with optimism for the future, a deeper understanding of the condition, and daily-use instruments [13]. 

A DBT skills module called “Walking The Middle Path” [25] addresses adolescents' and their families' specific problems and skill deficits. This program has reduced conflict and made relationships “closer” and “warmer” by teaching participants to practice validation, mindfulness, and acceptance of self and others. These skills are shown to help decrease emotional arousal, thus reducing the intensity of conflict, emotional dysregulation, and maladaptive behaviors. A cohort study [9] that involved at-risk samples of adolescent girls and their mothers revealed positive dyadic affective behaviors (i.e., satisfaction and positive escalation) and positive maternal affective behaviors (i.e., supportive/validating conduct, communication skills, and positive affect) were linked to decreases in the girls' BPD severity scores over time. A faster decline in daughters' BPD severity scores was specifically predicted by maternal communication abilities, capacity to foster autonomy, positive affect, and support/validation. These findings not only support the role of the mother-daughter context as a crucial barrier against the progression of BPD severity scores throughout adolescence but also shows that positive caregiver behavior, which could potentially be implemented by family intervention or family therapy, could positively influence the symptoms and severity of BPD in adolescent patients. A single-blind randomized controlled trial from Norway [26] of 77 adolescents with recent and repetitive self-injurious behavior compared DBT for adolescents (DBT-A), which contained one weekly session of multifamily skills training and family therapy sessions with enhanced usual care (EUC) showed that DBT-A is more effective than EUC in reducing suicidal and self-injurious behavior, suicidal thoughts, and depressive symptoms. DBT-A is also found to reduce the use of emergency services and hospital admissions. The reduction of self-harm is retained at further follow-ups at one year [27] and three years [28]. 

Family therapy is used as part of MBT programs as well. A randomized controlled trial in the United Kingdom [29] compared mentalization-based treatment for adolescents (MBT-A), which was designed as a 12-month intervention program including weekly individual sessions and monthly family therapy (MBT-F), with treatment as usual (TAU) that prescribes evidence-based interventions according to diagnostic criteria. This study has shown that MBT-A, which contained family therapy, was significantly more effective than TAU in reducing self-harm, depression, and borderline personality features and contributed these effects to positive change in mentalizing and improved interpersonal functioning. In this study, significantly fewer participants in the TAU group (33%) than in the MBT-A group (63%) received family-based intervention in the MBT-A group, which may potentially show the effect of family intervention in the treatment of BPD patients. 

Training programs specially targeted at caregivers of adolescent BPD patients have also shown a positive impact on the quality of care delivered to them, contributing to their well-being. A program adapting DBT-A to the Spanish national health system [30] used a 12-session psychoeducational group for parents of adolescents with emotional dysregulation for families not taking part in the interventions directed at adolescents themselves, such as individual therapy, skills group, and email therapy. Parents are educated to raise awareness of their emotions, cultivate an understanding of emotional dysregulation, and develop communication and crisis management skills. Clinical observation shows a decrease in self-harm behaviors and an increase in emotion regulation skills in the patients, while patients and family members reported better parent-child communication. A quantitative analysis investigating the impact of Family Connections Program, a 12-week training program originally created for caregivers of adolescent BPD patients in Canada [31], shows favorable outcomes, reducing caregiver burden and parenting stress, as well as child behavioral concerns, affect, mastery, and coping. Another quantitative study of the same program [32] uncovered that it improved caregivers’ ability to handle their children’s mental health challenges, impacted their intra- and interpersonal spheres, and improved the program itself. 

The efficacy of family intervention in managing adolescent BPD has been demonstrated by studies summarized in Table 2.

**Table 2:** Summary of Studies Conducted on the Use of Family Intervention in Adolescent BPD BPD = borderline personality disorder; ATraPA = actions for the treatment of adolescent personality; ATraPA-TAI = actions for the treatment of adolescent personality, intensive outpatient treatment; ATraPA-FAL = actions for the treatment of adolescent personality, families on the border; DBT = dialectical behavioral therapy; DBT-A = dialectical behavior therapy for adolescents; EUC = enhanced usual care; RCT = randomized controlled trial; MBT-A = mentalization-based treatment for adolescents; TAU = treatment as usual; NNT = number needed to treat; CI-BPD = childhood interview for DSM-4-TR borderline personality disorder; SIPA = Stress Index for Parents of Adolescents

Authors	Intervention format	Design & Setting	Participant information	Outcome measures	Outcome
Whalen et al. (2014) [9]	Adolescents' Axis I and II symptoms were evaluated using semistructured clinical interviews. Adolescents and their mothers engaged in a structured conflict discussion task	Cohort study/outpatient	110 adolescent females aged 16 years and their biological mothers	International Personality Disorders Examination, The Revised Interactional Dimensions Coding System	Positive maternal and dyadic affective behaviors were associated with more rapid rates of BPD severity scores declining over time
Mayoral et al. (2020) [30]	ATraPA-TAI: group-based intervention containing skills group, individual therapy, and email therapy. ATraPA-FAL: 12-session psychoeducational group for parents. Alternatives Group: weekly 1-hour session with inpatient adolescents with self-injurious behavior running in the healthcare system for 7 years	Observational study/outpatient	Adolescents aged 13-17 presenting with emotional dysregulation and their families referred within the region of Madrid, Spain	Not applicable	Decrease in the number of self-injurious behaviors, increase in emotion regulation skills, better parent-child communication
Boritz et al. (2021) [31]	Caregivers of adolescent BPD patients underwent 12 weekly 90-minute sessions in a skills training group. Individual therapy for the patients was not conducted	Quantitative analysis/outpatient	94 caregivers of adolescent BPD patients	Primary outcomes: Burden Assessment Scale, Stress Index for Parents of Adolescents. Secondary outcomes: The Child Behaviour Checklist, The Family Experience Interview Schedule, The Pearlin Mastery Scale, The DBT-Ways of Coping Checklist, The Grief Scale	Primary outcomes (Caregiver Burden and Parenting Stress), as well as secondary outcomes (child behavioral concerns, affect, mastery, and coping), improved. All outcome measures, except the SIPA (Parent Domain), showed statistically significant improvements over time
Sheikhan et al. (2021) [32]	Caregivers took part in semi-structured interviews at the end of 12 weekly 90-minute sessions in the form of a skills training group. Individual therapy for patients not conducted	Quantitative analysis/outpatient	13 caregivers of adolescent BPD patients	Interviews were audio-recorded, transcribed verbatim, de-identified and entered into qualitative analysis software for analysis	Three major themes regarding caregiver’s experience were identified: (a) caregiver's ability to manage their youth's mental health challenges was increased; (b) caregiver's inter-and intra-personal spheres were enhanced; (c) caregivers' experience led to proposed improvements to the program
Rathus et al. (2015) [25]	A demographic form (Treatment Acceptability Scale) and open-ended assessment administered following completion of the four or five-week module	Qualitative analysis/outpatient	50 participants recruited from three DBT programs in New York	Treatment Acceptability Scale scale, Child and Adolescent Mental Health Satisfaction Scale, DBT Skills Rating Scale for Adolescents	Middle path was regarded as helpful, interesting, and relevant. Overall both parents and adolescents rated it as most helpful. Both adolescents and parents considered conflicts reduced and relationships “closer” and “warmer”
Mehlum et al. (2014) [26], (2016) [27], (2019) [28]	19-week DBT-A: Weekly individual therapy, multifamily skills training, family therapy sessions, and telephone coaching EUC: Standard care enhanced by 1 weekly treatment session/psychodynamic/cognitive behavioral therapy combined with psychopharmacological treatment as needed	RCT with 1-year and 3-year follow-up/outpatient	N = 77, DBT = 39, EUC=38, aged 12-18, 1-year follow-up: N = 75, DBT = 38, EUC = 37, 3-year follow-up: N = 71 DBT = 37, EUC = 34	Suicidal ideation questionnaire, Moods and feelings questionnaire, Beck hopelessness scale, borderline symptom list, number of self-reported self-harm episodes, Montgomery—Asberg depression rating scale—baseline and 19 weeks, hospital admissions and emergency department visits, child behavior checklist, children’s global assessment scale, lifetime parasuicide count interview, suicide intent scale	DBT-A outperformed EUC in reducing suicidal and self-injurious behavior, suicidal ideation, and depressive symptoms. No significant group differences in borderline symptoms and hopelessness post-DBT. First follow-up (2016): Significant between-group differences in self-harm for the DBT group, but not in suicidal ideation, global functioning, hopelessness, borderline and depressive symptoms. Additional follow-up (2019): Significant group difference in frequency of self-harm episodes for the DBT-A group. Both groups remained on average at the same levels for suicidal ideation, depressive symptoms, hopelessness, borderline symptoms, and general functioning as at the first follow-up (2016). In patients who received DBT-A, receiving more than 3 months of follow-up care in the first year following completion was linked to better outcomes
Rossouw et al. (2012) [29]	1-year MBT-A: Weekly individual MBT-A sessions and monthly MBT-F sessions TAU: routine care by community-based adolescent mental health services	RCT with measurement at 3, 6, 9, and 12 months postrandomization/outpatient	N = 80, MBT = 40, TAU = 40, F/M = 68/12, aged 12-17 years old	Primary outcome: Risk-Taking and Self-Harm Inventory. Secondary outcomes: Mood and Feelings Questionnaire, Risk-taking scale, Borderline Personality Features Scale for Children, How I Feel questionnaire, Experience of Close Relationships Inventory	Primary outcome: Both groups demonstrated significant reductions in self-harm and risk-taking behavior. Self-harm scores were significantly lower for the MBT-A group at the 12-month point. Secondary outcome: Using the cutoff point of 8 at the Mood and Feelings Questionnaire for probable clinical depression, a significantly fewer percentage of participants of the MBT-A group scored in the clinical range than the TAU group at 9 months and at treatment end respectively (41% versus 70%, p = 0.03, NNT = 3.5, 95% CI 2.07 to 21.12) and (49% versus 68%, p = 0.08, NNT = –5.31, 95% CI –2.45 to 30.62)

Limitations

This study has several limitations. It was difficult to establish search criteria in order to locate relevant journals and studies, which appears to reflect the limited application of family therapy approaches and family intervention. Therefore, the designs and outcome measures of the studies included were heterogeneous, consisting of cohort studies, randomized controlled trials, and qualitative and quantitative studies. Due to the small sample sizes of the included studies, the conclusions cannot be generalized to the entire population. Family intervention was commenced as a part of other treatment modalities (DBT and MBT), which makes it difficult for accurate outcome evaluation. Still lacking are data evaluating unambiguous and consistent proof of the positive effect of family intervention alone. Retention of adolescents and their families in treatment long enough to provide effects was challenging, and adherence measures and follow-up assessments were lacking, causing difficulty in assessing long-term effects. Despite these limitations, results suggest that the usage of family intervention has a positive impact on the treatment and prognosis of adolescent BPD patients. As a result, this review appropriately acknowledges the limits of the studies while presenting the findings of a narrative synthesis. 

The engagement of families that may be dysfunctional is an important obstacle and dilemma for mental health services. Patients are hesitant to involve their families because they frequently have negative perceptions or prior negative experiences with them. In future research, the baseline function of the families participating needs to be taken into account, for it could impact the efficacy of the treatment. 

## Conclusions

This review’s purpose was to study the role of family interventions in treating adolescent patients with BPD. This is significant because families are an important part of adolescents’ life, and mistreatment plays a significant role in the pathogenesis of BPD. Most of the research combined family intervention with extant treatment methods such as DBT and MBT. It was demonstrated that family intervention has a promising scope in reducing self-harm, suicidal ideation, and depressive symptoms, improving emotion regulation, enhancing communication and interpersonal skills, and alleviating parenting stress and caregiver burdens in the studied population. It discovered limited evidence for the use and efficacy of family therapy approaches alone for adolescents with BPD in the general mental health field. Nevertheless, it suggests that family intervention in adolescent BPD patients may help recovery and provide patients and their families with the ability to manage the disorder's broader spectrum of problems, issues, and dynamics.

Future research can include trials comparing only family intervention with a control group; the study population should include a larger number of subjects. The baseline function for different families should be considered. Barriers to using family intervention should be identified and addressed. More research is needed to explore whether family involvement alone may be effective for both adolescents with BPD and their families. It would be beneficial to develop a clinical practice guideline for the implementation of a family systems approach by mental health service providers in order to enable a more consistent and practicable approach to engagement, referral, and follow-up that is setting-specific.
